# Public perceptions of the COVID-19 pandemic management in Bangladesh: a qualitative exploration

**DOI:** 10.12688/f1000research.28333.1

**Published:** 2021-03-02

**Authors:** Taufique Joarder, Muhammad N.B. Khaled, Mohammad A.I. Joarder

**Affiliations:** 1 Public Health Foundation, Bangladesh, Dhaka, Bangladesh; 2 International Food Policy Research Institute, Dhaka, Bangladesh; 3 Kumudini Women’s Medical College Hospital, Tangail, Bangladesh

**Keywords:** COVID-19, Pandemic, Resilience, Health policy and systems research, Bangladesh

## Abstract

**Background:** Since the emergence of the COVID-19 outbreak, Government of Bangladesh (GoB) has taken various measures to restrict virus transmission and inform the people of the situation. However, the success of such measures largely depends on a positive public perception of the government’s ability to act decisively and the transparency of its communication. We explored public perceptions of pandemic management efforts by the Bangladeshi health sector decision-makers in this study.

**Methods:** As this qualitative research was conducted during the COVID-19 pandemic, data was gathered through seven online mixed-gender focus group discussions involving 50 purposively selected clinicians and non-clinicians.

**Results:** The study participants concurred that, from the outset, decision-makers failed to engage the right kind of experts, which resulted in poor pandemic management that included imposing lockdown in periphery areas without arranging patient transport to the center, declaring certain hospitals as COVID-19 dedicated without preparing the facilities or the staff, and engaging private hospitals in care without allowing them to test the patients for COVID-19 infection. Several participants also commented on ineffective actions on behalf of the GoB, such as imposing home quarantine instead of institutional, corruption, miscommunication, and inadequate private sector regulation. The perception of the people regarding service providers is that they lacked responsiveness in providing treatment, with some doctors misleading the public by sharing misinformation. Service providers, on the other hand, observed that decision-makers failed to provide them with proper training, personal protective equipment, and workplace security, which has resulted in a high number of deaths among medical staff.

**Conclusions:** The Bangladeshi health sector decision-makers should learn from their mistakes to prevent further unnecessary loss of life and long-term economic downturn. They should adopt a science-based response to the COVID-19 pandemic in the short term while striving to develop a more resilient health system in the long run.

## Introduction

In December 2019, an unknown pneumonia-like disease appeared in Wuhan, China, but rapidly spread across the globe, prompting the World Health Organization (WHO) to label it as Coronavirus Disease 2019 (COVID-19) ^1^. On 30 January 2020, the WHO declared a Public Health Emergency of International Concern (PHEIC) followed by pandemic declaration on 11 March 2020 ^2^. In Bangladesh, the first case of COVID-19 infection was detected on 8 March, resulting in closure of educational institutions on 16 March. Following the first death on 18 March, in an attempt to contain the spread of the disease, the Government of Bangladesh (GoB) declared a ‘general holiday’ from 26 March to 4 April, which was repeatedly extended until 9 April, 14 April, 25 April, 5 May, 16 May, and 30 May. Despite these measures, COVID-19 infections continued to increase, but lockdown (which was formally termed by the government as ‘general holidays’) was withdrawn on 31 May ^3^. The number of confirmed COVID-19 cases in Bangladesh exceeded 100,000 on 18 June, and the upward trend continued throughout summer, with 200,000 cases recorded on 18 July, 300,000 on 26 August, and 400,000 on 27 October ^4^. By November 2020, Bangladesh ranked 24 ^th^ and 20 ^th^ in the world with respect to the total number of cases and deaths, respectively. At one point, Bangladesh ranked 3 ^rd^ in terms of the number of new cases per day, but given that the country’s test rate (15,863 per million inhabitants) is among the lowest in the world (it is the second-lowest after Afghanistan among its South Asian neighbors) these figures are likely to be much higher ^5^.

**Figure 1.**
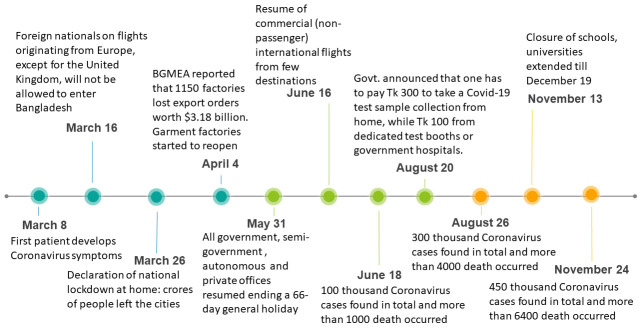
COVID-19 timeline in Bangladesh.

Pandemic response in Bangladesh is guided by the Infectious Diseases (Prevention, Control, and Elimination) Act 2018, which places the Directorate General of Health Services (DGHS) as the central coordinating and responsible body for COVID-19 response. Institution of Epidemiology, Disease Control, and Research (IEDCR) is the main scientific body to provide technical guidance and support for screening at the point of entry, and is in charge of imposing quarantine, managing contact tracing, and conducting initial testing (which was later contracted out to other government and a few private laboratories), while also providing forecasting and surveillance services, and the overall outbreak response ^6^. This agency was highly criticized for monopolizing all the COVID-19 tests in the first three weeks following the detection of the first case in a country of 180 million inhabitants. It was also blamed by the medical community for severe shortages of personal protective equipment (PPE) and tests, due to which many health professionals refused to provide care to infected individuals ^7^. To address this issue, from 3 April, approvals for additional test facilities were gradually issued, initially in the public and later in private facilities, totaling to 117 on 18 November ^8^. However, even though GeneXpert equipment was already available for tuberculosis test, this antigen-based rapid test was only made available for COVID-19 test by the government in July 2020 ^9^. Major events related to the COVID-19 pandemic in Bangladesh are shown in Figure 1.

Since the COVID-19 outbreak, the GoB has taken various measures to inform the public of the situation and restrict the transmission of the infection. However, available evidence indicates that success of such measures largely depends on a positive public perception of government’s ability to manage pandemics effectively, as well as foster multi-stakeholder cooperation ^10^, garner social order within the population ^10, 11^ and ensure good governance ^12, 13^. In this context, timely and transparent communication is essential ^14, 15^, as is involvement of technical and health experts in decision-making ^10, 16^. Gathering and sharing information on newly infected individuals and their contacts (as a part of contact tracing activities) is an important pandemic response activity, which can only be effective if the general public trusts the relevant agencies and service providers ^10, 14, 16^. When people have a positive perception of the health system, they are more likely to adhere to any measures imposed to protect public health ^11, 16, 17^. Thus, COVID-19 response requires adaptive leadership capable of making bold decisions and passing timely regulations based on the most recent scientific evidence, which is impossible without a positive perception of or trust on decision-makers and all pertinent stakeholders, including general public ^18^.

Although the epidemiologic features of SARS-COV-2 virus ^19, 20^, its clinical manifestations in different patient groups ^21, 22^, and its molecular characteristics ^23– 25^, as well as health systems response ^26^, economic and social consequences ^27– 29^, and public attitudes toward the measures implemented in Bangladesh have been investigated ^30– 32^, public perceptions of pandemic management efforts by the responsible bodies have never been studied. Motivated by the work of Bigdeli *et al*., we decided to explore the public perceptions of COVID-19 pandemic management in Bangladesh by focusing on the relationships between (1) people and the decision-makers (or the larger health system governance), (2) people and the service providers (only physicians were covered in this study), and (3) service providers and decision-makers ^33^. Findings yielded by this qualitative study will help decision-makers in introducing new or revising existing measures to allow service providers to better respond to the pandemic and increase public trust in the health system.

## Methods

### Study design

To gather the data for this qualitative study, seven focus group discussions (FGDs) were conducted as a part of which participants’ perceptions of the COVID-19 pandemic management in Bangladesh were explored.

### Ethical considerations

All the respondents provided verbal informed consent. All ethical principles were adhered to. The research was reviewed and approved by the Ethical Review Committee of the Public Health Foundation, Bangladesh (Reference number: 02/2020). During the pandemic, it was nearly impossible to gather all the participants of the FGDs and collect written consent. One may argue that it might be collected through digital signature. However, not all the participants were technologically well-equipped and trained. For convenience and treating all the participants’ consent in the same manner, we collected verbal consent. The Ethical Review Committee (ERC) approved this procedure.

### Study site, study population and participant selection

The study was promoted via a Google Forms link circulated across social media and email databases. First, the Google Forms link was circulated on 19 May among the participants of a webinar on health system trust, organized by a youth organization, the United Nations Youth and Students Association of Bangladesh (UNYSAB). The link was further circulated across social media and email databases, requesting expression of interest to participate in the study. The email databases of the members of the Public Health Foundation Bangladesh and the UNYSAB have been used in this regard. For circulating the form, the link along with a request to fill-up the form, was posted in the network of social media groups of researchers, health professionals, and university-based organizations.

Then, from the list of all the interested persons, the participants were purposively selected such that the FGD participants could be broadly classified into clinicians (graduate students with medical or dental background pursuing degrees in public health at a private university; renowned public health experts with a medical background; and clinicians practicing medicine or dentistry) and non-clinicians (undergraduate students pursuing non-medical degrees such as management, marketing, botany, business, and pharmacy, etc. at a public university; undergraduate students pursuing public health degrees at a public university; undergraduate students pursuing degrees in food and nutrition at a public university; and different professionals such as executives, trainers, managers, and coordinators of public and private organizations).

### Data collection

Prior to commencing the FGDs, we developed a discussion guide in Bangla (see Extended data ^34^), which was pilot tested in a session where a large group of health professionals and university students were present, organized by the UNYSAB, and adjusted in terms of issues addressed and the pattern of the language, where necessary. The goal was to focus discussions on the topics pertinent to this investigation, i.e., participants’ perceptions of COVID-19 pandemic management by the health sector decision-makers and the service providers, the implications of the actions (or lack thereof) taken by the decision-makers and service providers, and suggestions for improvements in pandemic management strategies.

Once a sufficient number of participants of both genders was recruited, seven FGDs (which were recorded through the video conferencing software Google Meet) were conducted between 15 and 17 June 2020, each involving 6−10 participants.

The FGDs were moderated by the first author (male), who has a doctorate in public health and is a health policy and systems researcher with experience and expertise in qualitative research methods. As a researcher in public health, he knew the participants in the FGD’s with the health professionals and these participants knew him as well. The second author (male), trained in economics with a Master’s degree and experienced in qualitative research, assisted in notetaking. He, however, had no prior engagement with any of the participants. Each FGD lasted 60−105 minutes and was conducted in Bangla, the native language of the respondents and the researchers. The research interests were explicitly explained to the participants, but the floor was open and no leading discussion points were initiated.

### Data analysis

Prior to content analysis—chosen due to the scarcity of existing literature on pandemic management in Bangladesh—the FGDs were transcribed by the research team ^35^. For the diversity of the role among the FGD participants working in the health sector, data and observations were unique. In the FGD’s conducted among the university students, data saturation took place. Thematic analysis commenced with listening to the recordings and reading the transcripts, which allowed a coding schema to be developed in Microsoft Excel (version: Professional Plus 2016) based on the questions asked, noting the first impressions followed by labelling the text segments by newly emerging codes. Next, similar-meaning codes were merged and sorted into broader categories. To substantiate the emerging themes, appropriate excerpts from the FGDs were identified. In order to increase validity, the first and second author independently coded the dataset, seeking input from the third author in case of any disagreement.

## Results

### Background characteristics of the focus group participants

In total, 50 individuals (28 males and 22 females, aged 19−75 years) took part in seven FGDs ( Table 1). Four of these FGDs were held with individuals with a non-clinical background (n = 28) and the remaining three capture the views of clinicians (n = 22). Nearly half of the respondents had training in public health.

**Table 1.** Characteristics of the focus group discussion (FGD) participants.

FGD number	Group characteristics	Number of respondents	Age range in years	Male/ female	Clinical or non- clinical background
FGD-1	Undergraduate students pursuing degrees in management, marketing, botany, business, and pharmacy at a public university	6	19−21	5/1	Non-clinical
FGD-2	Graduate students with medicine or dentistry as their undergraduate background pursuing public health degrees at a private university	6	25−34	0/6	Clinical
FGD-3	Undergraduate students pursuing public health degrees at a public university	9	21−26	4/5	Non-clinical
FGD-4	Undergraduate students pursuing food and nutrition degrees at a public university	6	22−25	0/6	Non-clinical
FGD-5	Different professionals such as executives, trainers, managers, and coordinators of public and private organizations	7	24−28	6/1	Non-clinical
FGD-6	Renowned public health experts with medical background	7	45−75	5/2	Clinical
FGD-7	Practicing clinicians with either medical or dentistry background	9	28−67	8/1	Clinical

### Participants’ perceptions of the health system decision-makers

Participants’ perceptions regarding health systems decision-makers have been presented under three sub-themes: their perceptions regarding the preparatory phase of the pandemic (not appointing the appropriate professionals, leading to incorrect management steps), coordination (indecisions stemming from incoordination), and actions by the health sector decision-makers (miscommunication, poor regulation).

Perceptions regarding preparation

For years, the Bangladeshi health system has been undermined by budget shortages, lack of quality services, high out-of-pocket payments, unregulated private sector, and a highly centralized secondary or tertiary care. These issues hindered the pandemic response, as the right persons were not placed in the right positions at the outset, as explained by a professor of public health:

*“An epidemic is a public health emergency; it is neither clinical nor an administrative issue. So, we must see this problem through the public health lens. We* [epidemiologists] *already know what to do to control an epidemic. … We the public health professionals should be given the flexibility that we are free to do whatever is needed for the country, not something that just pleases the political leadership.”* [FGD-6, renowned public health experts, clinical background]

Failure to engage the right professionals in the decision-making resulted in a rapidly escalating public health crisis, as the testing capacity, medical equipment and PPE provisions in the health centers were lacking. Study participants also concurred that the crisis was exacerbated by not instituting subsistence allowance for the poor before imposing lockdown (denoted by the GoB as ‘general holiday’), by failing to allow sufficient time for families to prepare for shop closures, and for increasing uncertainty by extending lockdown on a weekly basis. In addition, the health system actors wasted critical time by initially conducting tests at a single government facility before allowing private centers, although few in number, to engage in testing and provide COVID-19 care. Non-government organizations, and National Tuberculosis Control Program of the government had access to antigen-based rapid test, GeneXpert machines, which were utilized at a much later stage of the pandemic. As one student of public health explained:

*“Government is allowing, although lately, private diagnostic centers to perform COVID-19 tests. But there are many other machines which are not yet being utilized.”* [FGD-2, graduate students of public health at a private university, clinical background]

Perceptions regarding coordination

Several FGD participants commented on lack of coordination which manifested through imposing lockdown in periphery areas of the country without arranging for the patients to be transported to the centrally located health facilities, declaring several hospitals as COVID-19 dedicated institutions without providing the required resources, and involving private hospitals in care provision without allowing them to test patients for COVID-19 on admission. Additionally, they were of view that lockdown was terminated due to the upcoming major Muslim religious holiday—the Eid—even though the outbreak was not showing any signs of abating. This decision was reached despite objections from the leading public health experts. Reflecting on these issues, a renowned public health expert remarked:

*“The civil administration must coordinate with the health system people at district and sub-district levels to ramp up the response against COVID-19. Success cannot be achieved without a tactful decentralization, involving different relevant ministries.”* [FGD-6, renowned public health experts, clinical background]

According to one FGD participant, lack of coordination contributed to the rapid spread of the disease among the low-income garment employees of Bangladesh:

*“BGMEA* [national trade organization of garment manufacturers] *ordered garment workers to return to Dhaka to save their jobs. After they returned by thousands, braving the coronavirus infection, BGMEA declared that the factories will not open. They* [BGMEA] *never consulted public health experts or health department. On the other hand, the police announced that they will not allow anyone to enter Dhaka. The innocent workers were caught between a rock and a hard place due to the lack of coordination between different departments.”* [FGD-2, graduate students of public health at a private university, clinical background]

Perceptions regarding actions taken by the health sector decision-makers

Several actions by the health sector decision-makers were openly criticized by the study participants. They were particularly critical of the decision to impose home rather than institutional quarantine at the beginning of the pandemic, even though intimate Bangladeshi culture is not conducive to home quarantine. They were also of view that point-of-entry screening was weak, and blamed widespread corruption for PPE shortages, purchases of sub-standard equipment and mismanagement of relief materials. These issues, along with miscommunication, were frequently discussed in media. Several participants also criticized the decision to disguise lockdown as a ‘general holiday’ in order to reduce panic, while failing to allow the residents enough time to prepare. This was aptly surmised by one participant, who noted:

*“When they* [government] *say it is a ‘general holiday’ instead of ‘lockdown’, people confuse it with something festive. That’s why we saw people going to Cox’s Bazar* [a popular tourist destination] *for tourism purposes. Some of my friends even got married during this period, taking advantage of the ‘general holiday’.”* [FGD-3, undergraduate students of public health at a public university, non-clinical background]

Poor regulation was another complaint voiced by many participants, who were of view that it caused rapid escalation in prices of essential goods due to panic buying, while permitting uncontrolled advertisement and sales of unproven COVID-19 medicines (e.g., hydroxychloroquine, ivermectin, remdesivir, etc.). One participant commented on the proliferation of unauthorized and even fake testing centers that were providing false COVID-19-negative certifications:

*“Today, I saw in the news that someone who had been found corona negative here was found positive after landing in Japan. So, when things like these happen, our trust is compromised. These instances may even adversely affect our foreign relations.”* [FGD-5, service holders of different professions, non-clinical background]

### Participants’ perceptions of the service providers

Participants’ perceptions regarding health service providers include lack of responsiveness of the providers, spreading misinformation, colluding in corruption.

When the pandemic started, some newspapers alleged that, fearing for their own safety, some doctors were refusing to provide service to COVID-19 patents, or were not responsive enough while providing treatment. As one public university student explained:

*“In hospitals, especially the government hospitals, doctors don’t care about the patients. Doctors should not only provide clinical care, but also explain the disease, talk to the patient with respect, and provide more time.”* [FGD-1, undergraduate students of different departments at a public university, non-clinical background]

Some doctors were also accused of spreading misinformation through social and mainstream media. In a video that was rapidly disseminated across social media, one doctor confidently claimed that coronavirus would go away in the summer, while another respected senior doctor openly advertised unproven medicines on TV. These actions were condemned by a participant:

*“I found many of my doctor friends posting about different treatments for COVID-19. I think this may confuse and mislead people, as different doctors are saying different things.”* [FGD-2, graduate students of public health at a private university, clinical background]

Since the COVID-19 pandemic began, a doctor has been found colluding with someone guilty of running unauthorized testing centers ^36^. Some were found promoting unproven medicines, and providing false certificates of COVID-19 negativity. One medicine (hydroxychloroquine) was initially included into the national treatment guidelines, only to be subsequently removed following the WHO’s warning about its ineffectiveness. A public health expert attributed these decisions to the vested interest of some clinicians serving on the technical committee in promoting certain treatments:

*“Some* [doctors] *are saying plasma therapy is the solution, some are promoting different other drugs like hydroxychloroquine even though the WHO is saying there is no specific treatment for COVID-19.”* [FGD-6, renowned public health experts, clinical background]

### Perceptions of the service providers about the decision-makers

Service providers’ perceptions regarding health systems decision-makers include leaving them unprepared and untrained in the face of the pandemic, not recognizing their sacrifices and the lack of workplace security stemming from COVID-19 mismanagement. 

Doctors that took part in this investigation felt that the health system decision-makers failed to prepare them adequately to combat COVID-19 effectively. They complained about lack of training, absence of treatment guidelines, PPE and equipment shortages, as well as inadequate food and logistic support while on duty. On this, a doctor that has been on COVID-19 duty since the pandemic outbreak said:

*“Doctors did not receive proper training or treatment guidelines, only online training, nothing on triage, how to handle indoor patients, no idea about treatment guideline, nothing on donning and doffing of PPE, or mental stress management. I feel like swimming in an unfathomable sea, without proper training.”* [FGD-7, practicing clinicians, clinical background]

These issues resulted in a very high number of deaths among Bangladeshi doctors, which further undermined healthcare providers’ trust in the health system. Despite their sacrifices, they were not granted prioritized testing or healthcare, while also experiencing delays in salary payments. As a result, many doctors lacked motivation, as explained by one participant:

*“Government did not clarify direction regarding who would get the motivation package. Some doctors did not even receive their regular salary. This demoralized the doctors. … I know several young doctors who are saying that, if they are assigned COVID-19 duty, they will simply resign.”* [FGD-6, renowned public health experts, clinical background]

Several doctors expressed concerns over workplace security, as Bangladeshi people take out their dissatisfaction over the health system inadequacies on the doctors. To highlight the growing violence which resulted in a death of a colleague, many doctors stopped telemedicine services which they were previously offering benevolently to combat the COVID-19 crisis. A physician engaged in COVID-19 response said:

*“Decision-makers should make the work environment of the doctors safe in such a way that they themselves would confidently send their own children for treatment. Many of them* [health sector decision-makers] *have children who are doctors. I personally know several of them who are forbidding their children to serve in COVID-19 units, because there is no security there.”* [FGD-7, practicing clinicians, clinical background]

## Discussion

The findings yielded by this qualitative study indicate that several problems emerged as a consequence of failure to engage the right kind of experts in managing the pandemic. As a result of poor decision-making, the Bangladeshi health system was inadequately prepared to respond to the COVID-19 outbreak, as evident in the negative perception of our participants regarding the service providers especially in terms of quality of care they provided, and misinformation some of them shared in the social and mainstream media. Service providers also complained about lack of training, PPE, equipment, motivational packages, and workplace security.

The finding that the Bangladeshi health system failed to engage the right experts in the right positions is supported by several news articles and reports covering this topic. The Government of Bangladesh formed a 17-member National Technical Advisory Committee (NTAC) on 19 April 2020, more than a month after the first COVID-19 case was detected in the country. In the interim, most of the pandemic control efforts were entrusted to bureaucrats or administrators, many of whom lacked expertise or experience in health, let alone pandemic management. It is also worth noting that only three members of the NTAC had a public health career track ^37^. This issue was further compounded on 21 April, when the government assigned 64 top bureaucrats to supervise and coordinate relief distribution activities in 64 districts of Bangladesh ^38^ without seeking input or technical leadership from public health professionals. A policy analysis on the human resources for health in Bangladesh revealed that the DGHS is principally managed by the clinicians at the expense of public health experts. The same applies to the Ministry of Health and Family Welfare level, which comprises of members drawn from other ministries often unrelated to the health sector ^39^. Given that such administrative approach is not conductive to pandemic management, lessons can be learned from Switzerland, Georgia, and New Zealand and other countries where science-based public health strategies have been proven highly effective ^40^.

Since doctors are often seen as the face of a health system, people blame them for any inadequacies in care delivery despite considerable sacrifices most doctors have made throughout the pandemic. So far, around 3,000 doctors in Bangladesh have contracted the virus and more than 100 have died due to COVID-19 ^41^. The negative perceptions regarding the service providers have been widely reported in Bangladeshi media ^42^, which were attributed to poor communication skills and inadequate responsiveness (i.e., addressing the social needs of the patients such as being treated with friendliness, respect, information, trust, and sensitivity) in recent academic studies ^43– 45^.

Service providers’ claims that inadequate training, PPE and equipment shortages are the main cause of their grievances have also been documented in other studies from Bangladesh. In a study conducted from 9 to 14 April 2020, Islam and colleagues examined the frontline health workers’ perceptions and opinions on their personal safety while attending COVID-19 patients. Their findings show that 29% of the participating doctors lacked training on PPE use, 18% lacked training on COVID-19 case management, and 11% of the respondents did not receive any PPE ^46^. Several news reports also highlighted the logistics issues related to food, lodging and transport provision for doctors working in COVID-19 dedicated hospitals ^47^.

### Policy recommendations

When managing any health crisis, a science-based professional response is necessary and must involve relevant experts such as public health professionals, including infectious disease epidemiologists, health policy and systems experts, medical anthropologists, health economists, and health communication experts; laboratory scientists, including virologists, microbiologists, biochemists, and lab technicians; and relevant clinicians, including physicians, nurses, and paramedics. In the long run, however, a separate public health track, which is currently absent in the Bangladeshi health sector, must be implemented ^39^. Service providers should be trained and directed to provide high-quality and efficient services with good quality and responsiveness ^48, 49^, while their legitimate demands should also be duly addressed.

### Research implications

Since this study did not capture the perspectives of health decision-makers, it would be beneficial to conduct further investigations into health system governance incorporating their perspectives. Quantitative research should also be conducted to explore patients’ views on the responsiveness of the service providers, as well as service providers’ perspectives on their own safety and experiences during the COVID-19 pandemic.

### Limitations

The main limitation of this study stems from the use of online FGDs, which resulted in a sample that might not reflect the socioeconomic and demographic characteristics of the Bangladeshi population (as those without internet connectivity, or lower educational and socioeconomic status would be unable to respond to the Google Forms link or partake in online discussions). Consequently, the findings reported here cannot be generalized beyond the specific context in which the study was conducted. Second, it is worth noting that the first author was a COVID-19 patient at the time this study was conducted, which could potentially bias the qualitative analysis. However, every effort was made to reduce this risk through data triangulation ^50^, and by engaging multiple research team members in data coding and interpretation.

## Conclusions

Bangladesh experienced several local disease outbreaks over the past several years ^51– 54^ as well as a dengue epidemic in 2019 ^55^, but due to their lower magnitude compared to the COVID-19 pandemic, the need for a comprehensive overhauling of the health systems has not been felt so deeply before. Low- and middle-income countries like Bangladesh are particularly vulnerable to pandemics due to their week governance and limited health system preparedness ^56^. This article focused on the public perceptions of the pandemic management efforts by the health system actors, as the aim was to help the decision-makers and service providers in implementing more effective public health protection measures.

The main contribution of this investigation stems from highlighting the need to engage the right kind of experts in the right places at the outset of pandemic management efforts. It is further noted that public trust can be improved by being more transparent in official communications, while addressing the needs of service providers. These findings can help decision-makers revise their policies in order to prevent a longer-term loss of life and economic downturn. In addressing the COVID-19 pandemic or any future public health crisis, a science-based professional response is indispensable.

## Data availability

### Underlying data

Harvard Dataverse: Extended data: “Public perceptions of the COVID-19 pandemic management in Bangladesh: a qualitative exploration”, https://doi.org/10.7910/DVN/CJZKJH ^34^.

This project contains de-identified transcripts in Bangla.

### Extended data

Harvard Dataverse: Extended data: “Public perceptions of the COVID-19 pandemic management in Bangladesh: a qualitative exploration”, https://doi.org/10.7910/DVN/CJZKJH ^34^.

This project contains the following extended data:

-    Discussion guide used in the FDGs in both Bangla and English.

Data are available under the terms of the Creative Commons Zero “No rights reserved” data waiver (CC0 1.0 Public domain dedication).
