# Supplementary material for: Urban educated group's perceptions of the COVID-19 pandemic management in Bangladesh: a qualitative exploration
Source: F1000Res. 2021 Apr 20;10:170. Originally published 2021 Mar 2. [Version 2] doi: 10.12688/f1000research.28333.2 (PMC8444153; doi:10.12688/f1000research.28333.2)
Supplement: Peer review history

# Peer review: Reviewer response for version 2

**DOI:** 10.5256/f1000research.56082.r92028

**Published:** 2021-09-15

**Authors:** Shafiun Nahin Shimul

**Affiliations:** 1 Institute of Health Economics, University of Dhaka, Dhaka, Bangladesh

The study attempted to understand public perceptions of the pandemic management efforts of Bangladesh using qualitative tools.  Although most of the findings of this study are very well known, the study has made those well-known issues evident. I think this is a significant contribution in that sense. In addition, the policy recommendations emanated from this study appear to be relevant.

Is the work clearly and accurately presented and does it cite the current literature?

Yes

If applicable, is the statistical analysis and its interpretation appropriate?

Yes

Are all the source data underlying the results available to ensure full reproducibility?

Yes

Is the study design appropriate and is the work technically sound?

Yes

Are the conclusions drawn adequately supported by the results?

Yes

Are sufficient details of methods and analysis provided to allow replication by others?

Yes

Reviewer Expertise:

Health Economics, Public Finance, Behavioral Economics, Public Policy and Public Health

I confirm that I have read this submission and believe that I have an appropriate level of expertise to confirm that it is of an acceptable scientific standard.

# Peer review: Reviewer response for version 2

**DOI:** 10.5256/f1000research.56082.r83637

**Published:** 2021-05-18

**Authors:** Theresa Hoke

**Affiliations:** 1 FHI 360, Durham, NC, USA

The authors have thoroughly and successfully addressed the reviewers' comments. This is a sound qualitative investigation that makes an important contribution to the literature. This real-time scientific documentation of the current pandemic will provide important lessons learned for future crises.

Is the work clearly and accurately presented and does it cite the current literature?

Yes

If applicable, is the statistical analysis and its interpretation appropriate?

Not applicable

Are all the source data underlying the results available to ensure full reproducibility?

Yes

Is the study design appropriate and is the work technically sound?

Yes

Are the conclusions drawn adequately supported by the results?

Yes

Are sufficient details of methods and analysis provided to allow replication by others?

Yes

Reviewer Expertise:

Public health; implementation research; health systems research.

I confirm that I have read this submission and believe that I have an appropriate level of expertise to confirm that it is of an acceptable scientific standard.

# Peer review: Reviewer response for version 1

**DOI:** 10.5256/f1000research.31340.r80584

**Published:** 2021-03-26

**Authors:** Manuela Villar Uribe

**Affiliations:** 1 World Bank Group, Washington, DC, USA

Thank you for inviting me to review this insightful manuscript. It comes at a very important time when discussions on lessons learned will help strengthen the health system in the short and medium term. The article is well written and presents results clearly and adequately. 

 I only have three comments that could help strengthen the manuscript: 

1. The participants in the focus groups are primarily students, health workers or health system experts. I suggest the results and title of the manuscript are framed more clearly to ensure the reader understands that the views presented are not actually representative of the general public but are rather the views of the health community in Bangladesh. Presenting and discussing the findings in light of the representation that the participants provide of a public health and medical population is also extremely valuable and better targeted for use in system strengthening activities to come: trust and coordination with and among medical professionals probably ought to have been a key component to the COVID response.
2. I am not sure I understand why you have two sections in the results where you outline issues related to decision-makers. I suggest creating a joint section for those two sub-sections where you can highlight the role they played and allow the reader to foresee areas of improvement at the decision-maker level. 
3. I am particularly interested by policy recommendations and would encourage the authors to strengthen that section further by outlining recommendations in line with the results that are presented: what recommendations stem from the views on coordination, preparation, decision-makers, etc?

Is the work clearly and accurately presented and does it cite the current literature?

Yes

If applicable, is the statistical analysis and its interpretation appropriate?

Not applicable

Are all the source data underlying the results available to ensure full reproducibility?

Yes

Is the study design appropriate and is the work technically sound?

Yes

Are the conclusions drawn adequately supported by the results?

Partly

Are sufficient details of methods and analysis provided to allow replication by others?

Yes

Reviewer Expertise:

Public health, Primary Health Care, Measurement of Quality of Care, Health Policy

I confirm that I have read this submission and believe that I have an appropriate level of expertise to confirm that it is of an acceptable scientific standard, however I have significant reservations, as outlined above.

# Response

**Published:** 2021-03-28

**Authors:** Taufique Joarder

**Affiliations:** Public Health Foundation, Bangladesh, Bangladesh

We have found the comments to be very thoughtful. We will address the comments and revise our manuscript soon, along with a point-by-point response. We thank the reviewer for taking the time to review and allow us to improve our manuscript.

# Response

**Published:** 2021-04-11

**Authors:** Taufique Joarder

**Affiliations:** Public Health Foundation, Bangladesh, Bangladesh

The participants in the focus groups are primarily students, health workers or health system experts. I suggest the results and title of the manuscript are framed more clearly to ensure the reader understands that the views presented are not actually representative of the general public but are rather the views of the health community in Bangladesh. Presenting and discussing the findings in light of the representation that the participants provide of a public health and medical population is also extremely valuable and better targeted for use in system strengthening activities to come: trust and coordination with and among medical professionals probably ought to have been a key component to the COVID response.

**Response:** Thanks for the suggestion regarding the title of the manuscript. Since not all the participants belong to the health community, we edited the title a bit differently, which now reads as follows: "Urban educated groups' perceptions of the COVID-19 pandemic management in Bangladesh: a qualitative exploration." We have now also edited the results such that the readers can understand whose views were presented and how prevalent the sentiment was across the FGDs.

I am not sure I understand why you have two sections in the results where you outline issues related to decision-makers. I suggest creating a joint section for those two sub-sections where you can highlight the role they played and allow the reader to foresee areas of improvement at the decision-maker level.

**Response:** This study was motivated by the work of Bigdeli *et al*. (2020)[1]. In this article, the authors proposed the Health Systems Governance Framework, adapted from the World Development Report 2004. This framework discusses relationships between three different spheres: 1) Between the Policymakers and Providers, 2) Between Providers and the People, and 3) Between the People and the Policy-Makers. We mentioned this in the last paragraph of the Background section. We explored the public perceptions of COVID-19 pandemic management in Bangladesh by focusing on the relationships between (1) people and the decision-makers (or the larger health system governance), (2) people, and the service providers (only physicians were covered in this study), and (3) service providers and decision-makers. Aligned with the objective of the article, the three sub-sections under the Result section have been organized. Creating the joint section will undermine the alignment with the research objectives.

 [1] Bigdeli M, Rouffy B, Lane BD, Schmets G, Soucat A. Health systems governance: the missing links. BMJ Global Health. 2020 Aug 1;5(8):e002533.

I am particularly interested by policy recommendations and would encourage the authors to strengthen that section further by outlining recommendations in line with the results that are presented: what recommendations stem from the views on coordination, preparation, decision-makers, etc?

**Response:** Thank you for this suggestion. While reviewing the manuscript, we clearly saw the weaknesses in the Recommendations section. We have now revised the entire section according to the advice of the reviewer.

# Peer review: Reviewer response for version 1

**DOI:** 10.5256/f1000research.31340.r80588

**Published:** 2021-03-24

**Authors:** Theresa Hoke

**Affiliations:** 1 FHI 360, Durham, NC, USA

The authors have conducted a timely study investigating an intriguing aspect of the COVID-19 pandemic: public perceptions of pandemic management efforts by Bangladeshi health sector leaders. They applied sound qualitative methods to answer the research question. Three types of relationships are considered: people and decision-makers; people and providers; and providers and decision-makers.

 The authors are encouraged to consider the following points as they revise the paper to make it even stronger.

Substantive issues

 p.4 first column seems to indicate physicians were the only providers included, yet p.4 column 2 mentions clinicians practicing dentistry. In Bangladesh, are the latter considered physicians?

 In reporting results, provide some indication of how prevalent the sentiment was across the focus groups. For example, was the issue mentioned in just one group, or in about half the groups, or in all the groups? Similarly, if there was a strong sentiment within a group, echoed by many participants, mention that. In reading the following text, for example, it was not obvious whether this was information derived directly from the FGDs, or if it was the authors’ perspective: “For years, the Bangladeshi health system has been undermined by budget shortages, lack of quality services, high out-of-pocket payments, unregulated private sector, and a highly centralized secondary or tertiary care.” On p.6, “Several FGD participants commented on lack of coordination….” Were these participants in a single group, or across several groups?

 On p.6: “One participant commented on the proliferation of unauthorized and even fake testing centers….” Then on p.7: “Since the COVID-19 pandemic began, a doctor has been found colluding with someone guilty of running unauthorized testing centers”. Can these matters be reported together?

 p.7: “Service providers’ perceptions regarding health systems decision-makers include leaving them unprepared and untrained in the face of the pandemic, not recognizing their sacrifices and the lack of workplace security stemming from COVID-19 mismanagement.” This text reads like a summary of findings rather than results coming directly from the FGDs. Consider moving that text to the Discussion.

 p.7 The authors are encouraged to carefully consider how news articles are used to support research findings. How much confidence can be placed in the fundamental accuracy of news articles? Additionally, is it possible that news articles support what study participants say because study participants have been influenced by what they heard in the press?

 Limitations: Undergraduate students represent a major portion of the non-clinician participants. This is a legitimate approach, but this particular group does bring a special perspective, since university students tend to be younger and of higher socio-economic status than the population at large. Any potential bias could be mentioned in the Limitations section.

Minor issues

 Check wording in the following places:

 Figure 1: March 26— Scores of people. June 16: Resumption of commercial…

 Replace FGD’s with FGDs in a couple of places.

 Take another look at this wording: p.5 “For the diversity of the role among the FGD participants working in the health sector, data and observations were unique.” The meaning is not completely clear.

Is the work clearly and accurately presented and does it cite the current literature?

Yes

If applicable, is the statistical analysis and its interpretation appropriate?

Not applicable

Are all the source data underlying the results available to ensure full reproducibility?

Yes

Is the study design appropriate and is the work technically sound?

Yes

Are the conclusions drawn adequately supported by the results?

Yes

Are sufficient details of methods and analysis provided to allow replication by others?

Yes

Reviewer Expertise:

Public health; implementation research; health systems research.

I confirm that I have read this submission and believe that I have an appropriate level of expertise to confirm that it is of an acceptable scientific standard, however I have significant reservations, as outlined above.

# Response

**Published:** 2021-04-11

**Authors:** Taufique Joarder

**Affiliations:** Public Health Foundation, Bangladesh, Bangladesh

p.4 first column seems to indicate physicians were the only providers included, yet p.4 column 2 mentions clinicians practicing dentistry. In Bangladesh, are the latter considered physicians?

**Response:** In Bangladesh, MBBS (Bachelor of Medicine and Bachelor of Surgery) and BDS (Bachelor of Dental Surgery) are considered equivalent degrees, and both the holders of MBBS and BDS degrees are treated similarly at the professional level. For example, job circulars in the public sector specify MBBS and BDS degrees for applicants while referring to them as 'doctors' in general. The regulatory authority for providing medical practice license in Bangladesh, the Bangladesh Medical and Dental Council (BMDC), recognizes MBBS and BDS degree-holders as 'doctors,' and restrict others (for example the Sub-Assistant Community Medical Officers, village doctors, etc.) from using a 'Dr.' before their name to indicate their clinical qualification.

In reporting results, provide some indication of how prevalent the sentiment was across the focus groups. For example, was the issue mentioned in just one group, or in about half the groups, or in all the groups? Similarly, if there was a strong sentiment within a group, echoed by many participants, mention that. In reading the following text, for example, it was not obvious whether this was information derived directly from the FGDs, or if it was the authors’ perspective: “For years, the Bangladeshi health system has been undermined by budget shortages, lack of quality services, high out-of-pocket payments, unregulated private sector, and a highly centralized secondary or tertiary care.” On p.6, “Several FGD participants commented on lack of coordination….” Were these participants in a single group, or across several groups?

**Response:** This is a very useful suggestion. We have now provided some indication of how prevalent the sentiment was across the FGDs.

On p.6: "One participant commented on the proliferation of unauthorized and even fake testing centers…." Then on p.7: "Since the COVID-19 pandemic began, a doctor has been found colluding with someone guilty of running unauthorized testing centers". Can these matters be reported together?

**Response:** Sure. We have now removed the statement from p.7 to p.6 to present them together.

p.7: "Service providers' perceptions regarding health systems decision-makers include leaving them unprepared and untrained in the face of the pandemic, not recognizing their sacrifices and the lack of workplace security stemming from COVID-19 mismanagement." This text reads like a summary of findings rather than results coming directly from the FGDs. Consider moving that text to the Discussion.

**Response:** Based on the initial review comment by the F1000Research reviewer, we added a summary of findings at the beginning of each sub-section. The part indicated in the comment is, in fact, the summary of findings. If deemed unacceptable by the reviewer, we have to discuss this issue again with the editorial team and resolve it.

p.7 The authors are encouraged to carefully consider how news articles are used to support research findings. How much confidence can be placed in the fundamental accuracy of news articles? Additionally, is it possible that news articles support what study participants say because study participants have been influenced by what they heard in the press?

**Response:** Various authentic media outlets in Bangladesh widely circulated the news articles reported in this manuscript. Given the nature of the issues reported, we think that the news articles substantiate the respondents' expressed views. We agree that there is a possibility of the participants being influenced by the press. However, this issue, in our opinion, will not invalidate our findings because the study's objective was to explore the public perceptions of COVID-19 pandemic management in Bangladesh, and many factors, including the media, naturally influence public perceptions.

Limitations: Undergraduate students represent a major portion of the non-clinician participants. This is a legitimate approach, but this particular group does bring a special perspective, since university students tend to be younger and of higher socio-economic status than the population at large. Any potential bias could be mentioned in the Limitations section.

**Response:** Agreed. We have now added this issue in the Limitations, as suggested.

 Check wording in the following places:

Figure 1: March 26— *Scores* of people. June 16: *Resumption* of commercial…

Replace FGD's with FGDs in a couple of places.

**Response:** Thanks for identifying these errors. We have revised the text.

Take another look at this wording: p.5 "For the diversity of the role among the FGD participants working in the health sector, data and observations were unique." The meaning is not completely clear.

**Response:** We revised the sentence as follows: "For the diversity of the role among the FGD participants working in the health sector, data saturation was not achieved." We hope the meaning is clear now.
